# 16GT: a fast and sensitive variant caller using a 16-genotype probabilistic model

**DOI:** 10.1093/gigascience/gix045

**Published:** 2017-06-15

**Authors:** Ruibang Luo, Michael C. Schatz, Steven L. Salzberg

**Affiliations:** 1 Department of Computer Science, Johns Hopkins University, Baltimore, MD 21218, USA; 2 Center for Computational Biology, McKusick-Nathans Institute of Genetic Medicine, Johns Hopkins University School of Medicine, Baltimore, MD 21218, USA; 3 Departments of Biomedical Engineering and Biostatistics, Johns Hopkins University, Baltimore, MD 21218, USA

**Keywords:** variant calling, Bayesian model, SNP calling, indel calling

## Abstract

16GT is a variant caller for Illumina whole-genome and whole-exome sequencing data. It uses a new 16-genotype probabilistic model to unify single nucleotide polymorphism and insertion and deletion calling in a single variant calling algorithm. In benchmark comparisons with 5 other widely used variant callers on a modern 36-core server, 16GT demonstrated improved sensitivity in calling single nucleotide polymorphisms, and it provided comparable sensitivity and accuracy for calling insertions and deletions as compared to the GATK HaplotypeCaller. 16GT is available at https://github.com/aquaskyline/16GT.

## Background

Single nucleotide polymorphisms (SNPs) and insertions and deletions (indels) that occur at a specific genome position are interdependent; i.e., evidence that elevates the probability of 1 variant type should decrease the probability of other possible variant types, and the probability of all possible alleles should sum to 1. However, widely used tools such as GATK’s UnifiedGenotyper [1] and SAMtools [2] use separate models for SNP and indel detection. The model for SNP calling in these 2 tools is nearly identical: both assume all variants are biallelic (i.e., exactly 2 haplotypes are present) and use a probabilistic model allowing for 10 genotypes: AA, AC, AG, AT, CC, CG, CT, GG, GT, TT. For indel calling, the GATK UnifiedGenotyper uses a model from the Dindel's variant caller [3], while SAMtools’ model is from BAQ [4].

## Findings

In order to detect SNPs and indels with a unified approach, we developed a new 16-genotype probabilistic model and its implementation, named 16GT. Building on an idea first introduced in Luo et al. [5], 16GT uses an empirically improved model and is the first publicly available implementation. Using X and Y to denote the indels with the highest (X) and second highest (Y) support, we add 6 new genotypes (AX, CX, GX, TX, XX, and XY) to the traditional 10-genotype probabilistic model. The 6 new genotypes include: (i) 1 homozygous indel (XX); (ii) 1 reference allele plus 1 heterozygous indel (AX, CX, GX, TX); (iii) 1 heterozygous SNP plus 1 heterozygous indel (AX, CX, GX, TX, reusing the genotypes in ii); and (iv) 2 heterozygous indels (XY). We exclude the 5 possible combinations AY, CY, GY, TY, and YY because X has higher support than Y. By unifying SNP and indel calling in a single variant calling algorithm, 16GT not only runs 4 times faster, but also demonstrates improved sensitivity in calling SNPs and comparable sensitivity in calling indels to the GATK HaplotypeCaller.

Posterior probabilities of these 16 genotypes are calculated using a Bayesian model *P*(*L*|*F*)∝*P*(*F*|*L*)*P*(*L*), where *L* is an assumed genotype. *F* refers to the observation of the 6 alleles (A, C, G, T, X, Y) at a given genome position. *P*(*L*) is the prior probability of the genotype, *P*(*F*|*L*) is the likelihood of the observed genotype, and *P*(*L*|*F*) is the posterior probability of the genotype. The resulting genotype *L_max_* is assigned to the genotype with the highest posterior probability. The distance between the highest posterior probability and the second highest posterior probability is used as a quality metric in 16GT, along with some other metrics introduced by GATK (GATK, RRID: SCR_001876) [1].

### Calculating the probability of an observation F given the genotype L

To test how well an observation fits the expectation of different genotypes, we use a 2-tailed Fisher exact test *P* and use the resulting *P*-value as the goodness of fit. When calculating the likelihood of a homozygous genotype, ideally we expect 100% single allele support from the observation. For example, consider genotype “AA”:

\begin{equation*}
P( {F{|^{\rm{'}}}{\rm{A}}{{\rm{A}}^{\rm{'}}}}) = {P_{hom}} \left( {{F_A}} \right) \times {P_e}\left( {{F_C},{F_G},{F_T},{F_X},{F_Y}} \right),
\end{equation*}

where *P_e_* is the probability of an erroneous base call.

For a heterozygous genotype, 50% support is expected for each allele in the genotype; e.g., consider “CG”:

\begin{equation*}
P( {F{|^{'}}C{G^{'}}}) = {P_{het}} \left( {{F_C},{F_G}} \right) \times {P_e}\left( {{F_A},\ {F_T},{F_X},{F_Y}} \right),
\end{equation*}

where

\begin{equation*}
{P_{hom}}\left( {{F_A}} \right) = \ P\left( {\begin{array}{@{}*{2}{c}@{}} {{F_A}}&F\\ {\left( {1 - {P_{err}}} \right)F}&F \end{array}} \right)
\end{equation*}

\begin{equation*}
{P_{het}} \left( {{F_C},{F_G}} \right) = \sqrt {\mathop \prod \limits_{i = C,G} P\left( {\begin{array}{@{}*{2}{c}@{}} {{F_i}}&F\\ {\left( {0.5 - {P_{err}}} \right)F}&F \end{array}} \right)} \
\end{equation*}

\begin{equation*}
{P_e}\left( {{F_A},{F_T},{F_X},{F_Y}} \right) = \ P\left( {\begin{array}{@{}*{2}{c}@{}} {{F_A} + {F_T} + {F_X} + {F_Y}}&F\\ {{P_{err}} \times F}&F \end{array}} \right)
\end{equation*}

\begin{equation*}
{F_s} = \mathop \sum \limits_{i = 1}^n f\left( {{Q_i},{M_i},s} \right)\ \ \ \ s\, \epsilon \left\{ {A,C,G,T,X,Y} \right\},
\end{equation*}

where *s* is the allele type, *n* is the number of reads supporting allele *s, Q_i_* is the base quality, and *M_i_* is the mapping quality. *f* is a function describing how *s, Q_i_*, and *M_i_* change the observation:

\begin{equation*}
f\ \left( {{Q_i},{M_i},s} \right) = \alpha \times \beta \times \gamma \left\{ {\begin{array}{@{}*{1}{l}@{}} {\alpha \ = \ 0\quad if\quad \ {M_i} = \ 0}\\ {\alpha \ = \ 1\quad if\quad {M_i} \ne 0}\\ {\beta \ = \ 0\quad if\quad {Q_i} < 10}\\ {\beta \ = \ 1\quad if\quad 10 \le {Q_i} < 13}\\ {\beta \ = \ 2\quad if\quad 13 \le {Q_i} < 17}\\ {\beta \ = \ 3\quad if\quad 17 \le {Q_i} < 20}\\ {\beta \ = \ 4\quad if\quad {Q_i} \ge 20}\\ {\gamma \ = \ 1\quad if\quad s\quad\epsilon \left\{ {A,C,G,T} \right\}}\\ {\gamma \ = \ 1.375\quad if\quad s\,\,\epsilon \left\{ {X,Y} \right\}} \end{array}.} \right.
\end{equation*}

The possible reasons for an observation that does not match the reference genome are (i) a true variant; (ii) an error generated in library construction; (iii) a base calling error; (iv) a mapping error; and (v) an error in the reference genome. Reasons (iii) and (iv) are explicitly captured in our model. For reasons (ii) and (v), we include 2 error probabilities, *P_s_* for SNP error and *P_d_* for indel error. We define *P_err_* as *P_s_+P_d_*, where *P_s_* and *P_d_* are set to 0.01 and 0.005, respectively. These 2 values were set empirically based on the observation that SNP errors are more common than indel errors in library construction and in the reference genome.

In addition, most short read aligners use a dynamic programming algorithm to enable gapped alignment, using a scoring scheme that usually penalizes gap opening and extension more than mismatch. Consequently, authentic gaps that occur at an end of a read are more likely to be substituted by a set of false SNPs or alternatively to get trimmed or clipped. Thus, we applied a coefficient γ to weight indel observations more than SNPs in order to increase the sensitivity on indels.

### Calculating the probability of the genotype L

Given (i) a known rate of single nucleotide differences between 2 unrelated haplotypes; (ii) a known rate of single indel differences between 2 unrelated haplotypes; and (iii) a known Transitions to Transversions ratio (Ti/Tv), the 16GT model's prior probabilities are calculated as shown in Table 1.

**Table 1:** P(L), Genotype prior probabilities for a reference allele “A”.

*L*	Zygosity	Number of SNPs	Number of indels	Number of transversions	Prior probability *P*(*L*)
AA	Hom.	–	–	0	1
GG	Hom.	1	0	2	θ/2 * ε * ε
CC, TT	Hom.	1	0	0	θ/2
AG	Het.	1	0	1	θ * ε
AC, AT	Het.	1	0	0	θ
CG, GT	Het.	2	0	1	θ * θ/2 * ε
CT	Het.	2	0	0	θ * θ/2
AX	Het.	0	1	0	ω
GX	Het.	1	1	1	ω * θ/2 * ε
CX, TX	Het.	1	1	0	ω * θ/2
XX	Hom.	0	1	0	ω/2
XY	Het.	0	2	0	ω * ω/2

Hom.: homozygous; Het.: heterozygous.

Given (i) a known rate θ of single nucleotide differences between 2 unrelated haplotypes; (ii) a known rate ω of single indel differences between 2 unrelated haplotypes; and (iii) a known Ti/Tv ε, transition is expected to occur more frequently than transversion under selective pressure. The default known rates for human genome are θ = 0.001, ω = 0.0001, ε = 2.1, where *ε* is set to the value for human and needs to be changed for other species.

## Results

We benchmarked 16GT with GATK UnifiedGenotyper, GATK HaplotypeCaller (GATK, RRID: SCR_001876) [1], Freebayes (FreeBayes, RRID: SCR_010761) [6], Fermikit [7], ISAAC (Isaac, RRID: SCR_012772) [8], and VarScan2 [9] using a set of very high-confidence variants developed by the Genome in a Bottle project for genome NA12878 (Coriell Cat# GM12878, RRID: CVCL_7526; version 2.19) (Additional File 1: Supplementary Note) [10]. The results are shown in Table 2 and as receiver operating characteristic curves in Supplementary Fig. S1.

**Table 2:** Benchmark comparisons between 16GT and 5 other variant callers on a dataset from the Genome in a Bottle project consisting of 787M read pairs (53-fold) from genome NA12878.

		SNP						Indel				
			FP						FP			
Caller	Time (minutes w/36 cores)	TP	Total	dbSNP 138	dbSNP 138%	TP in Omni 2.5	FN	TP	Total	dbSNP 138	dbSNP 138%	FN
16GT	121	2 663 179	5346	4220	79%	20/20	918	167 549	1462	944	65%	3180
UG	29	2 655 608	1639	563	34%	15/15	8489	163 839	624	546	88%	6890
HC	539	2 653 684	419	143	34%	4/4	10 413	168 444	1232	726	59%	2285
Freebayes	52	2 655 513	724	353	49%	11/14	8584	162 505	559	0	0%	8224
Fermikit	45	2 567 672	2036	509	25%	9/9	96 425	161 916	1996	1076	54%	8813
ISAAC	63	2 659 438	1115	586	53%	15/15	4659	158 642	1239	710	57%	12 087
VarScan2	136	2 658 358	1680	718	43%	10/10	5739	158 906	574	481	84%	11 823

FP: false positive; FN: false negative; HC: GATK HaplotypeCaller; UG: GATK UnifiedGenotyper.

For SNPs, 16GT produced the most true positive calls and the fewest false negative calls; i.e., it has the highest sensitivity and specificity among all tools. dbSNP version 138 also reported 79% of 16GT’s false positive calls, which is the highest among other callers. However, we should point out that the GIAB variant set is biased toward GATK because it was primarily derived from GATK-based analyses, as reported previously [11]. As an orthogonal test, we further assessed the false positive calls against a set of unbiased calls made by the Illumina Omni 2.5 SNP array (Additional File 1: Supplementary Note). Among the 5346 false positive calls for 16GT, 20 were covered by the Omni array, and all 20 (100%) had the correct genotype. Although limited by the small number of measurable alleles in the Illumina Omni 2.5 SNP array, only allowing us to reassess 20 “false positive” calls as true positives, the observation that all 20 genotypes out of the 20 covered alleles are correct suggests that a number of the remaining “false positive” calls are actually correct.

For indels, 16GT produced slightly fewer true positive calls and slightly more false negative calls than HaplotypeCaller, but less than half as many false negative calls as UnifiedGenotyper. dbSNP version 138 covered 65% of 16GT’s false positive indels. Further investigation into the 1462 false positive indels shows that 981 (67%) of them meet all 3 of the following criteria: (i) at least 3 reads supporting the variant; (ii) at least 1 read supporting both the positive and negative strands; and (iii) in over 80% of the reads that support the variant, there exists no other variant in its flanking 10 bp. This suggests that some of these “false positives” might be correct, although further experimental validation would be required to confirm this suggestion. Supplementary Fig. S2 shows 3 examples where the putative false positive from 16GT is likely to be correct.

## Conclusions

16GT is the firstly publicly available implementation using a 16-genotype probabilistic model for variant calling. Compared with local assembly based variant callers, 16GT provides better sensitivity in SNP calling and comparable sensitivity in indel calling. In the current implementation, 16GT can only be applied to germline variant detection. In the future, we will enhance 16GT to support multi-sample variant calling and GVCF output and to support somatic variant detection and extend the model to support variant calling in species with more than 2 haplotypes.

## Additional files

Additional File 1.docx

## Abbreviations

indel: insertions and deletions; SNP: single nucleotide polymorphism; Ti/Tv: Transitions to Transversions.

## Supplementary Material

GIGA-D-17-00091_Original-Submission.pdf

GIGA-D-17-00091_Revision-1.pdf

Response-to-Reviewer-Comments_Original-Submission.pdf

Reviewer-1-Report-(Original-Submission).pdf

Reviewer-2-Report-(Original-Submission).pdf

Additional File 1.docx

## Availability of source code and requirements

Project name: 16GT

Project homepage: https://github.com/aquaskyline/16GT

Archived version: https://github.com/aquaskyline/16GT/releases/tag/1.0

Operating system: Platform independent

Programming language: C++ and Perl

Other requirements: See GitHub page

License: GPLv3

Any restrictions to use by non-academics: None

## Availability of supporting data and materials

Snapshots of the code and data are available in the *GigaScience* repository, GigaDB [12], and are also available via the Code Ocean reproducibility platform [13].
