# Supplementary material for: 16GT: a fast and sensitive variant caller using a 16-genotype probabilistic model
Source: Gigascience. 2017 Jun 15;6(7):1–4. doi: 10.1093/gigascience/gix045 (PMC5570013; doi:10.1093/gigascience/gix045)
Supplement: GIGA-D-17-00091_Revision-1.pdf [file gix045_GIGA-D-17-00091_Revision-1.pdf]

Manuscript Click here to download Manuscript 16GT-GigaScience-2.docx

16GT: a fast and sensitive variant caller using a 16-genotype probabilistic model

Ruibang Luo[1,2,*], Michael C. Schatz[1,2], Steven L. Salzberg[1,2,3]

[1]Department of Computer Science, Johns Hopkins University

[2]Center for Computational Biology, McKusick-Nathans Institute of Genetic Medicine, Johns Hopkins University School of Medicine

[3]Departments of Biomedical Engineering and Biostatistics, Johns Hopkins University

Email addresses:

Ruibang Luo, rluo5@jhu.edu (ORCID: 0000-0001-9711-6533)

Michael C. Schatz, mschatz@jhu.edu (ORCID: 0000-0002-4118-4446)

Steven L. Salzberg, salzberg@jhu.edu (ORCID: 0000-0002-8859-7432)

*Corresponding Author

Abstract

16GT is a variant caller for Illumina whole-genome and whole-exome sequencing data. It uses a new 16-genotype probabilistic model to unify SNP and indel calling in a single variant calling algorithm. In benchmark comparisons with five other widely used variant callers on a modern 36-core server, 16GT demonstrated improved sensitivity in calling SNPs, and it provided comparable sensitivity and accuracy for calling indels as compared to the GATK HaplotypeCaller. 16GT is available at https://github.com/aquaskyline/16GT.

Keywords

Variant calling; Bayesian model; SNP calling; Indel calling

Background

Single nucleotide polymorphisms (SNPs) and insertions and deletions (indels) that occur at a specific genome position are interdependent; i.e., evidence that elevates the probability of one variant type should decrease the probability of other possible variant types, and the probability of all possible alleles should sum to 1. However, widely-used tools such as GATK's UnifiedGenotyper [1] and SAMtools [2] use separate models for SNP and indel detection. The model for SNP calling in these two tools is nearly identical: both assume all variants are biallelic (i.e., exactly two haplotypes are present) and use a probabilistic model allowing for 10 genotypes: AA, AC, AG, AT, CC, CG, CT, GG, GT, TT. For indel calling, the GATK UnifiedGenotyper uses a model from the Dindel's variant caller [3], while SAMtools' model is from BAQ [4].

Findings

In order to detect SNPs and indels with a unified approach, we developed a new 16-genotype probabilistic model and its implementation named 16GT. Building on an idea first introduced in Luo et al. [5], 16GT uses an empirically improved model and is the first publicly available implementation. Using X and Y to denote the indels with the highest (X) and second highest (Y) support, we add 6 new genotypes (AX, CX, GX, TX, XX and XY) to the traditional 10-genotype probabilistic model. The six new genotypes include: 1) one homozygous indel (XX); 2) one reference allele plus one heterozygous indel (AX, CX, GX, TX); 3) one heterozygous SNP plus one heterozygous indel (AX, CX, GX, TX, reusing the genotypes in 2); and 4) two heterozygous indels (XY). We exclude the 5 possible combinations AY, CY, GY, TY, YY because X has higher support than Y. By unifying SNP and indel calling in a single variant calling algorithm, 16GT not only runs 4 times faster, but also demonstrates improved sensitivity in calling SNPs and comparable sensitivity in calling indels to the GATK HaplotypeCaller.

Posterior probabilities of these 16 genotypes are calculated using a Bayesian model $P(L|F) \propto P(F|L)P(L)$, where L is an assumed genotype. F refers to the observation of the 6 alleles (A, C, G, T, X, Y) at a given genome position. $P(L)$ is the prior probability of the genotype, $P(F|L)$ is the likelihood of the observed genotype. and $P(L|F)$ is the posterior probability of the genotype. The resulting genotype L_{max} is assigned to the genotype with the highest posterior probability. The distance between the highest posterior probability and the second highest posterior probability is used as a quality metric in 16GT, along with some other metrics introduced by GATK (GATK , RRID:SCR_001876) [1].

Calculating the probability of an observation F given the genotype L

To test how well an observation fits the expectation of different genotypes, we use a two-tailed Fisher's Exact Test *P* and use the resulting *p*-value as the goodness of fit. When

calculating the likelihood of a homozygous genotype, ideally we expect 100% single allele support from the observation. For example, consider genotype ‘AA’:

$$P(F|'\mathrm{AA}') = P_{hom}(F_A) \times P_e(F_C, F_G, F_T, F_X, F_Y)$$

where P_e is the probability of an erroneous base call.

For a heterozygous genotype, 50% support is expected for each allele in the genotype, for example consider ‘CG’:

$$P(F|'CG') = P_{het}(F_C, F_G) \times P_e(F_A,\ F_T, F_X, F_Y)$$

where

$$P_{hom}(F_A) = P\begin{pmatrix} F_A & F \\ (1 - P_{err})F & F \end{pmatrix}$$

$$P_{het}(F_C, F_G) = \sqrt{\prod_{i=C,G} P\begin{pmatrix} F_i & F \\ (0.5 - P_{err})F & F \end{pmatrix}}$$

$$P_e(F_A, F_T, F_X, F_Y) = P\begin{pmatrix} F_A + F_T + F_X + F_Y & F \\ P_{err} \times F & F \end{pmatrix}$$

$$F_s = \sum_{i=1}^{n} f(Q_i, M_i, s) \quad s\epsilon\{A, C, G, T, X, Y\}$$

where s is the allele type, n is the number of reads supporting allele s, Q_i is the base quality, and M_i is the mapping quality. f is a function describing how s, Q_i and M_i change the observation:

$$f(Q_i, M_i, s) = \alpha \times \beta \times \gamma \begin{cases} \alpha = 0\ if\ M_i = 0 \\ \alpha = 1\ if\ M_i \neq 0 \\ \beta = 0\ if\ Q_i < 10 \\ \beta = 1\ if\ 10 \leq Q_i < 13 \\ \beta = 2\ if\ 13 \leq Q_i < 17 \\ \beta = 3\ if\ 17 \leq Q_i < 20 \\ \beta = 4\ if\ Q_i \geq 20 \\ \gamma = 1\ if\ s\epsilon\{A, C, G, T\} \\ \gamma = 1.375\ if\ s\epsilon\{X, Y\} \end{cases}$$

The possible reasons for an observation that does not match the reference genome are: 1) a true variant; 2) an error generated in library construction; 3) a base calling error; 4) a mapping error; and 5) an error in the reference genome. Reasons 3 and 4 are explicitly

captured in our model. For reasons 2 and 5, we include two error probabilities, P_s for SNP error and P_d for indel error. We define $P_{err}=P_s+P_d$, where P_s and P_d are set to 0.01 and 0.005, respectively. These two values were set empirically based on the observation that SNP errors are more common than indel errors in library construction and in the reference genome.

In addition, most short read aligners use a dynamic programming algorithm to enable gapped alignment, using a scoring scheme that usually penalizes gap opening and extension more than mismatch. Consequently, authentic gaps that occur at an end of a read are more likely to be substituted by a set of false SNPs or alternatively to get trimmed or clipped. Thus, we applied a coefficient γ to weight indel observations more than SNPs, in order to increase the sensitivity on indels.

Calculating the probability of the genotype L

Given 1) a known rate of single nucleotide differences between two unrelated haplotypes; 2) a known rate of single indel differences between two unrelated haplotypes; and 3) a known Transitions to Transversions ratio (Ti/Tv), the 16GT model's prior probabilities are calculated as shown in **Table 1**.

Table 1. P(L), Genotype prior probabilities for a reference allele 'A'.
Hom.: homozygous. Het.: heterozygous.

L	Zygosity	Number of SNPs	Number of Indels	Number of Transversions	Prior Probability *P*(*L*)
AA	Hom.	-	-	0	1
GG	Hom.	1	0	2	θ/2*ε*ε

CC, TT	Hom.	1	0	0	θ/2
AG	Het.	1	0	1	θ*ε
AC, AT	Het.	1	0	0	θ
CG, GT	Het.	2	0	1	θ*θ/2*ε
CT	Het.	2	0	0	θ*θ/2
AX	Het.	0	1	0	ω
GX	Het.	1	1	1	ω*θ/2*ε
CX, TX	Het.	1	1	0	ω*θ/2
XX	Hom.	0	1	0	ω/2
XY	Het.	0	2	0	ω*ω/2

Given 1) a known rate θ of single nucleotide differences between two unrelated haplotypes; 2) a known rate ω of single indel differences between two unrelated haplotypes; and 3) a known Transitions to Transversions ratio (Ti/Tv) ε, where transition is expected to occur more frequently than transversion under selective pressure. The default known rates for human genome are: $\theta = 0.001$, $\omega = 0.0001$, $\varepsilon = 2.1$, where ε is set to the value for human and needs to be changed for other species.

Results

We benchmarked 16GT with GATK UnifiedGenotyper, GATK HaplotypeCaller (GATK , RRID:SCR_001876) [1], Freebayes (FreeBayes, RRID:SCR_010761) [6], Fermikit [7], ISAAC (Isaac, RRID:SCR_012772) [8] and VarScan2 [9] using a set of very high-confidence variants developed by the Genome-in-a-bottle (GIAB) project for genome NA12878 (Coriell Cat# GM12878, RRID:CVCL_7526) [10] (version 2.19, Additional File 1: **Supplementary Note**). The results are shown in **Table 2** and as ROC curves in **Supplementary Figure 1**.

Table 2. Benchmark comparisons between 16GT and five other variant callers on a dataset from the Genome in a Bottle project consisting of 787M read pairs (53-fold) from genome NA12878. UG: GATK UnifiedGenotyper; HC: GATK HaplotypeCaller. FP: false positive, FN: false negative.

Caller	Time (minutes w/ 36 cores)	SNP						Indel				
		TP	FP				FN	TP	FP			FN
			Total	dbSNP 138	dbSNP 138 %	TP in Omni 2.5			Total	dbSNP 138	dbSNP 138 %	

16GT	121	2,663,179	5,346	4,220	79%	20/20	918	167,549	1,462	944	65%	3,180
UG	29	2,655,608	1,639	563	34%	15/15	8,489	163,839	624	546	88%	6,890
HC	539	2,653,684	419	143	34%	4/4	10,413	168,444	1,232	726	59%	2,285
Freebayes	52	2,655,513	724	353	49%	11/14	8,584	162,505	559	0	0%	8,224
Fermikit	45	2,567,672	2,036	509	25%	9/9	96,425	161,916	1,996	1,076	54%	8,813
ISAAC	63	2,659,438	1,115	586	53%	15/15	4,659	158,642	1,239	710	57%	12,087
VarScan2	136	2,658,358	1,680	718	43%	10/10	5,739	158,906	574	481	84%	11,823

For SNPs, 16GT produced the most true positive calls and the fewest false negative calls; i.e. it has the highest sensitivity and specificity among all tools. 79% of 16GT's false positive calls were also reported by dbSNP version 138, which is highest among other callers. However, we should point out that the GIAB variant set is biased towards GATK because it was primarily derived from GATK-based analyses, as reported previously [11]. As an orthogonal test, we further assessed the false positive calls against a set of unbiased calls made by the Illumina Omni 2.5 SNP array **(**Additional File 1: **Supplementary Note)**. Among the 5,346 false positive calls for 16GT, 20 were covered by the Omni array and all 20 (100%) had the correct genotype. Although limited by the small number of measurable alleles in the Illumina Omni 2.5 SNP array, only allowing us to reassess 20 ‘false positive' calls as true positives, the observation that all 20 genotypes out of the 20 covered alleles are correct suggests that a number of the remaining “false positive" calls are actually correct.

For indels, 16GT produced slightly fewer true positive calls and slightly more false negative calls than HaplotypeCaller, but less than half as many false negative calls as UnifiedGenotyper. 65% of 16GT's false positive indels were covered by dbSNP version 138. Further investigation into the 1,462 false positive indels shows that 981 (67%) of them meet all three of the following criteria: 1) at least three reads supporting the variant; 2) at least one read supporting both the positive and negative strands, and; 3) in over 80% of the reads that support the variant, there exists no other variant in its flanking 10bp. This suggests that some of these “false positives” might be correct, although further experimental validation

would be required to confirm this suggestion. **Supplementary Figure 2** shows three examples where the putative false positive from 16GT is likely to be correct.

Conclusions

16GT is the firstly publicly available implementation using a 16-genotype probabilistic model for variant calling. Compared with local assembly based variant callers, 16GT provides better sensitivity in SNP calling and comparable sensitivity in indel calling. In the current implementation, 16GT can only be applied to germline variant detection. In the future, we will enhance 16GT to support multi-sample variant calling and GVCF output, to support somatic variant detection and extend the model to support variant calling in species with more than two haplotypes.

Declarations

Abbreviations

GIAB: Genome-in-a-bottle; indel: insertions and deletions; SNP: single nucleotide polymorphism; Ti/Tv: Transitions to Transversions

Acknowledgements

We thank United Electronics Co. Limited for providing code samples for the bam2snapshot function.

Funding

This work has been supported by the U.S. National Institutes of Health under grants R01-HL129239 and R01-HG006677.

Availability of *source code and requirements*

Project name: 16GT

Project homepage: https://github.com/aquaskyline/16GT

Archived version: https://github.com/aquaskyline/16GT/releases/tag/1.0

Operating system: Platform independent

Programming language: C++ and Perl

Other requirements: See GitHub page

License: GPLv3

Any restrictions to use by non-academics: None

Availability of supporting data and materials

Snapshots of the code and data are available in the *GigaScience* GigaDB repository [12] and are also available via the Code Ocean reproducibility platform [13].

Authors' contribution

RL, MCS and SLS conceived the study. RL developed and implemented the 16GT algorithm and benchmarked 16GT with other variant callers. RL, MCS and SLS wrote the paper. All authors have read and approved the final version of the manuscript.

Competing interests

The authors declare that they have no competing interests.

References

1. McKenna A, Hanna M, Banks E, Sivachenko A, Cibulskis K, Kernytsky A, Garimella K, Altshuler D, Gabriel S, Daly M, DePristo MA: **The Genome Analysis Toolkit: a**

MapReduce framework for analyzing next-generation DNA sequencing data. *Genome Res* 2010, **20:**1297-1303.

2. Li H, Handsaker B, Wysoker A, Fennell T, Ruan J, Homer N, Marth G, Abecasis G, Durbin R, Genome Project Data Processing S: **The Sequence Alignment/Map format and SAMtools.** *Bioinformatics* 2009, **25:**2078-2079.
3. Albers CA, Lunter G, MacArthur DG, McVean G, Ouwehand WH, Durbin R: **Dindel: accurate indel calls from short-read data.** *Genome Res* 2011, **21:**961-973.
4. Li H: **Improving SNP discovery by base alignment quality.** *Bioinformatics* 2011, **27:**1157-1158.
5. Luo R, Wong YL, Law WC, Lee LK, Cheung J, Liu CM, Lam TW: **BALSA: integrated secondary analysis for whole-genome and whole-exome sequencing, accelerated by GPU.** *PeerJ* 2014, **2:**e421.
6. Garrison E, Marth G: **Haplotype-based variant detection from short-read sequencing.** *arXiv preprint arXiv:12073907* 2012.
7. Li H: **FermiKit: assembly-based variant calling for Illumina resequencing data.** *Bioinformatics* 2015**:**btv440.
8. Raczy C, Petrovski R, Saunders CT, Chorny I, Kruglyak S, Margulies EH, Chuang H-Y, Källberg M, Kumar SA, Liao A: **Isaac: ultra-fast whole-genome secondary analysis on Illumina sequencing platforms.** *Bioinformatics* 2013**:**btt314.
9. Koboldt DC, Zhang Q, Larson DE, Shen D, McLellan MD, Lin L, Miller CA, Mardis ER, Ding L, Wilson RK: **VarScan 2: somatic mutation and copy number alteration discovery in cancer by exome sequencing.** *Genome Res* 2012, **22:**568-576.
10. Zook JM, Chapman B, Wang J, Mittelman D, Hofmann O, Hide W, Salit M: **Integrating human sequence data sets provides a resource of benchmark SNP and indel genotype calls.** *Nat Biotechnol* 2014, **32:**246-251.
11. Chiang C, Layer RM, Faust GG, Lindberg MR, Rose DB, Garrison EP, Marth GT, Quinlan AR, Hall IM: **SpeedSeq: ultra-fast personal genome analysis and interpretation.** *Nat Methods* 2015, **12:**966-968.

12. Luo, R; Schatz, M, C; Salzberg, S, L (2017): **Supporting data for "16GT: a fast and sensitive variant caller using a 16-genotype probabilistic model"** GigaScience Database. http://dx.doi.org/10.5524/100316

13. Luo, R (2017): **16GT: a fast and sensitive variant caller using a 16-genotype probabilistic model [Source Code].** Code Ocean. http://dx.doi.org/10.24433/CO.0a812d9b-0ff3-4eb7-825f-76d3cd049a43

Click here to access/download
Supplementary Material
Additional File 1-2.docx

 Click here to download Personal Cover 16GT-Response to Reviewers.docx

Responses to
Comments of reviewers

16GT: a fast and sensitive variant caller using a 16-genotype probabilistic model

Ruibang Luo; Michael C Schatz; Steven L Salzberg

GIGA-D-17-00091

We appreciate the constructive comments from the editor and all the reviewers as well as their extra work on the evaluation of 16GT.

Editor

Comments:

1. One referee flags the comparisons you make, so please make sure there is sufficient comparisons and citation of the state-of-the-art in this field (e.g. its been highlighted on the pre-print that Scalpel, VarScan2, VarDict, Mutect2 and Strelka have not been included as benchmarks).

Response:
The comparison to VarScan2 has been added to the manuscript. Table 2 now comprises comparisons to six germline variant callers including two state-of-the-art callers named GATK-HC and Freebayes, and four other callers named, GATK-UG, Fermikit, ISAAC and VarScan2. VarDict, Mutect2 and Strelka are somatic variant callers, thus not compared to 16GT. Scalpel is an indel caller that doesn't detect SNPs, thus we did not compare it to 16GT. (Please note that one of us – MCS – is a co-author of Scalpel, so we know it well.)

Reviewer: 1

The authors present a new model that can call both SNPs and INDELs by expanding the number of possible allele states to 16. The paper is well written, the model is an interesting contribution, and the results are compelling. I would like to see a little more detail in a few sections of the paper.

The standard method for communicating the true positive / false negative trade off in variant calling is a ROC-style line plot. The shape of this curve can be insightful for readers who need place their experiments at different points along this plot depending on the particulars of their experiment. Since table 2 only reports a single point on that curve, the readers do not have this context. It is also not clear that these numbers represent comparable points along their curves.

Response:
We have added 7 ROC curves to our analysis, all shown in supplementary figure 1.

I don't understand why the proportion of false positives in dbSNP v138 is interesting when calling against NA12878 and why having a higher proportion in dnSNP v183 is better. I recognize that these are polymorphic sites, but what about that property is relevant to this analysis?

Response:
For a set of variants that are reported by any variant caller, previous studies show that variants found in dbSNP are much more likely to be true positives, because as you say these sites are known to be polymorphic in the population. Thus for any variant caller, a higher rate of overlap with dbSNP suggests a higher true positive rate. Similarly, if a "false positive" is also reported as a variant in dbSNP, previous studies suggest that it might not be false at all. This is why we mention how many of 16GT's "false" predictions are found in dbSNP - it suggests that some of them are true rather than false.

The idea has been utilized in multiple papers and presentations. Here I list and excerpt from three of them:

1) Screening the human exome: a comparison of whole genome and whole transcriptome sequencing, Cirulli et al., 2010. "SNVs called in the gDNA and cDNA were also compared with entries in dbSNP. It was found that 90% of the gDNA exonic SNVs corresponded to a dbSNP entry, while this was true of only 56% of the cDNA SNVs. However, a further breakdown revealed that 94% of the true positive cDNA SNVs corresponded to a dbSNP entry, while only 23% of the false positives did the same. The false negatives corresponded to dbSNP entries 89% of the time."
Link: https://dx.doi.org/10.1186%2Fgb-2010-11-5-r57

2) Sensitive detection of somatic point mutations in impure and heterogeneous cancer samples, Cibulskis et al., 2013. "Figure 3d: Somatic miscall error rate for true germ-line heterozygous single-nucleotide polymorphism sites by sequencing depth in the normal sample when the site is known to be variant in the population (in dbSNP) and previously unknown (not in dbSNP)"
Link: http://www.nature.com/nbt/journal/v31/n3/full/nbt.2514.html

3) Improving the Specificity of SNP Calls in the 1000 Genomes Project, Melgar et al., 2009. "Slide 7: SNPs that passed filter have 91% dbSNP. SNPs removed by filter have 33% dbSNP"
Link:
https://www.broadinstitute.org/files/shared/diversity/summerprogram/2009/mmelgar_presentation.pdf

We agree that the suggestion is relative not absolute. Thus, we highlighted in the manuscript that further experimental validation would be required to confirm this observation.

The model has several "empirically defined" parameters. It would be nice to describe this analysis so that users could modify the parameters for their own experiments. For example, the model will need to be retuned for long reads.

Response:
Empirically defined parameters include P_s: SNP error rate, P_d: Indel error rate, ϑ: rate of single nucleotide differences between two unrelated haplotypes, and ω: rate of single indel differences between two unrelated haplotypes. We found that the appropriate values for these appear to be stable across different species including human, thus we do not suggest that users modify them. For advanced users, we added comments to the code such that users can change the parameters easily. One thing that should change is ε, which is the transitions to transversions ratio, and we have now highlighted in the manuscript that ε is preset to the value for human and it needs to be changed for other species.

16GT does not appear to support multi-sample calling. I think the model presented here is good, but unless the software can handle many samples, or at least produce a GVCF, it may see little use.

Response:
We highlighted in the discussion that our next step to extend 16GT's functionality will include 1) supporting multi-sample variant calling and GVCF output, 2) supporting somatic variant detection, and 3)

extending the model to support variant calling in species with more than two haplotypes

- Ryan Layer, University of Utah

Reviewer: 2

Luo, R. etc described a new 16GT variant caller optimized for Illumina sequencing data that uses a new 16-genotype probabilistic model to unify SNP and indel calling. They demonstrated the improved sensitivity for SNPs and comparable accuracy for indels comparing to GATK HaplotypeCaller, using genome of NA12878 in GIAB project. 16GT more comprehensively models 16 genotypes to unify SNP and indel calling in the same algorithm. 16GT appears to be a useful alternative tool for analyzing germline sequencing using Illumina platform.
A few comments:

1. Need to emphasize that at least at the moment, 16GT can only be applied to germline sequencing using Illumina sequencing platform, and not appropriate for cancer genome sequencing, especially clinical cancer samples, where tumor cellularity varies greatly and not fit those models.

Response:
We now emphasize in the conclusion that, for now, 16GT can only be applied to germline variant detection. In the future, we will improve 16GT to support multi-sample variant calling and GVCF output, to support somatic variant detection and extend the model to support variant calling in species with more than two haplotypes.

2. Can authors comment on whether increased sensitivity of SNPs is due to incorporation of indels into the model, or are those additional SNPs called have indel as the 2nd allele?

Response:
16GT model performs better than the traditional 10-genotype model at a lower depth and when the authentic variant signals are mingled with noise of the other type. For example, investigation into the 3,710 Indels that detected by 16GT but missed in UnifiedGenotyper shows that 95.7% of them are lower than the mean depth and mingled with at least one mismatch. We observed additional SNPs with indels as the 2nd allele being called by 16GT than UnifiedGenotyper but not the HaplotypeCaller.

3. Can authors discuss the limitations of 16GT? What's the indel size limit? Should sex chromosomes be treated differently if gender is known?

Response:
The largest indel 16GT can detect is bounded by the aligner used for input generation. 16GT's algorithm has no limit on indel sizes. The 16GT implementation automatically detects the input gender and treats sex chromosomes differently.

4. I'm not keen to highlight better indel performance over GATK's UnifiedGenotyper, as it's known to be not a good indel caller, and not widely

used for indels nowadays.

Response:
We agree with the reviewer that UnifiedGenotyper is not widely used for indels after HaplotypeCaller has released. But since 16GT and UnifiedGenotyper are both Bayesian model based, a comparison between 16GT and UnifiedGenotyper can give readers some clues on how the better model improves the performance on indel calling. Note, also this is just one of the many comparisons we have included.

5. Given the run time in Table 2, I'm not sure "16GT ran faster" should be in the abstract.

Response:
We removed "ran faster" from the abstract.
