# Supplementary material for: 16GT: a fast and sensitive variant caller using a 16-genotype probabilistic model
Source: Gigascience. 2017 Jun 15;6(7):1–4. doi: 10.1093/gigascience/gix045 (PMC5570013; doi:10.1093/gigascience/gix045)
Supplement: Additional File 1.docx [file gix045_Supplement.docx]

16GT: a fast and sensitive variant caller using a 16-genotype probabilistic model

Ruibang Luo, Michael C. Schatz, Steven L. Salzberg

Supplementary Figure 1 2

Supplementary Figure 2 2

Supplementary Note: Evaluating the Methods 5

Download GIAB NA12878 truth dataset (Zook, et al., 2014) 5

Benchmarking using hapdip (Li, 2015) 5

Run 16GT 5

Run GATK UnifiedGenotyper 5

Run GATK HaplotypeCaller 6

Run Freebayes 6

Run Fermikit 6

Run ISAAC 7

Run VarScan2 7

Download Illumina Omni 2.5 and Evaluate SNPs (Genomes Project, et al., 2012) 7

Supplemental References 8

# Supplementary Figure 1

The ROC curves of the seven variant callers. 16GT achieves better true positive rate than other tools. The imcompleteness of the GIAB v2.19 database, which leads to a higher false positive rate in 16GT, is discussed in the maintext.

# Supplementary Figure 2

Three examples of a probably correct indel being classified as false positive.

a. 4bp deletion (CTCC, 1:50993512)


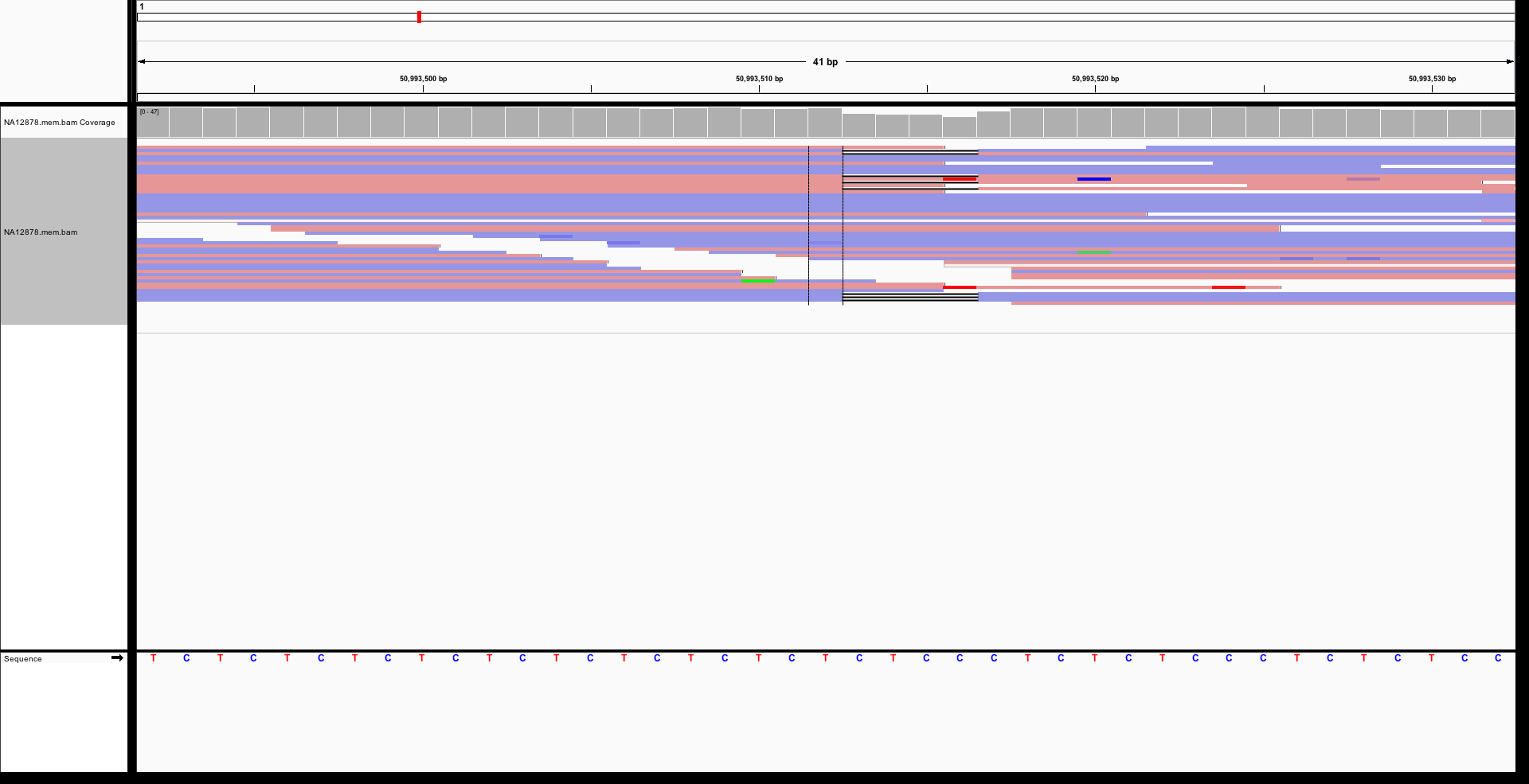


b. 4bp insertion (TTTA, 1:192067424)


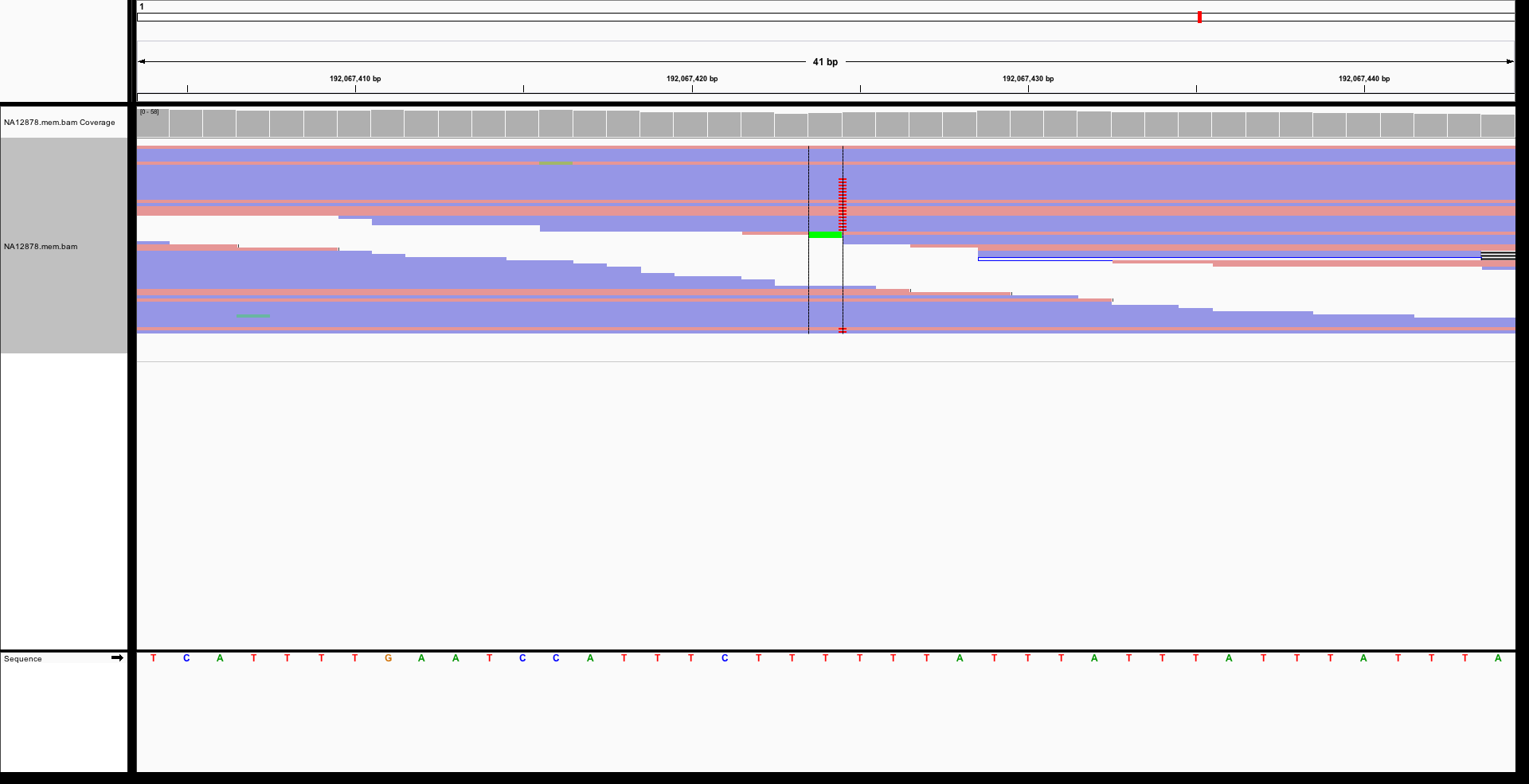


c. 8bp deletion (CCACACACA, 1:236058055)


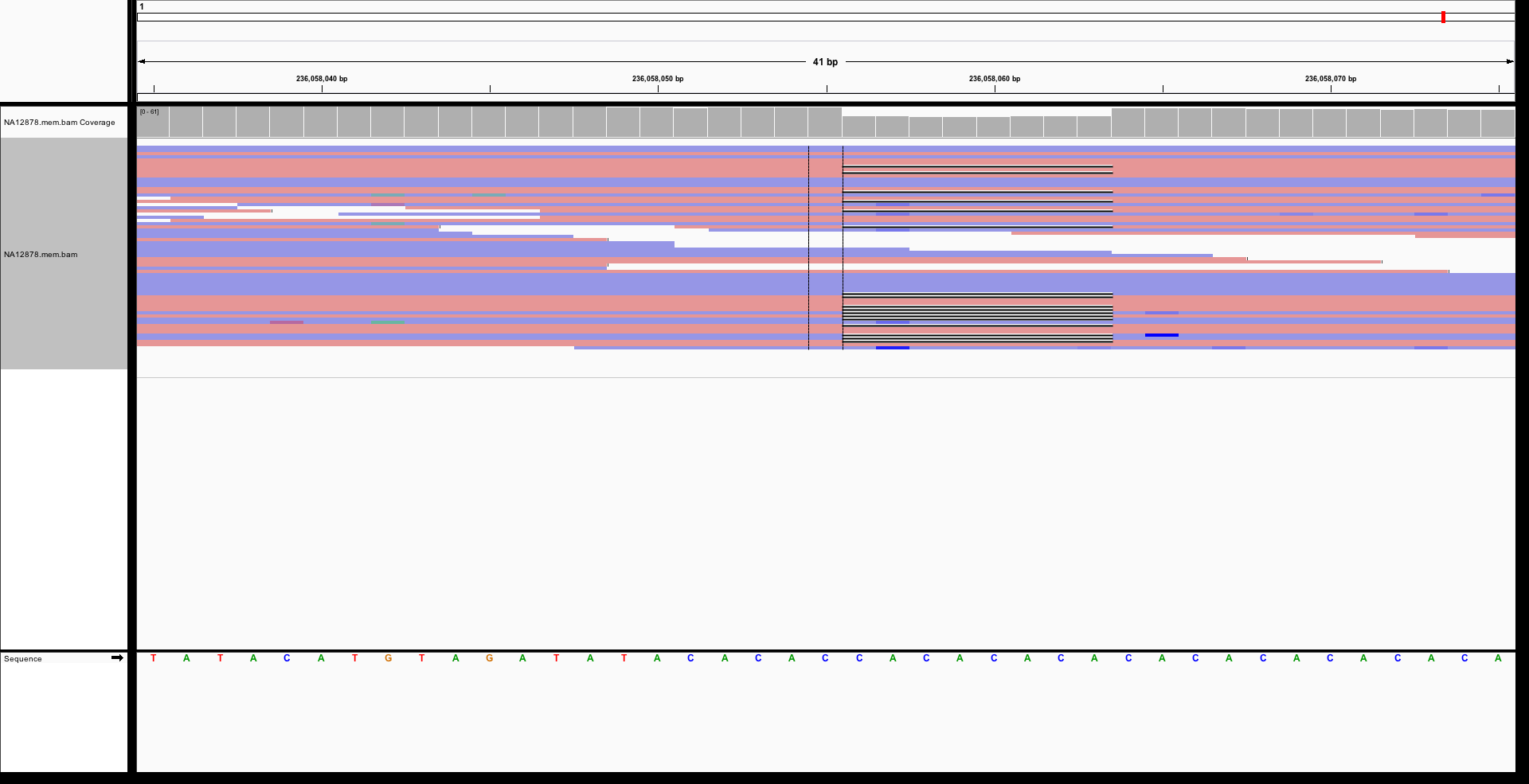


# Supplementary Note: Evaluating the Methods

## Download GIAB NA12878 truth dataset (Zook, et al., 2014)

# download the reference genome

wget ftp://ftp-trace.ncbi.nih.gov/1000genomes/ftp/technical/reference/phase2_reference_assembly_sequence/hs37d5.fa.gz

# download the alignments

wget ftp://hengli-data:lh3data@ftp.broadinstitute.org/hapdip/NA12878.mem.bam

# download GIAB-2.18 truth dataset

wget ftp://hengli-data:lh3data@ftp.broadinstitute.org/hapdip/GIAB/N+P.auto.bed.gz

wget ftp://hengli-data:lh3data@ftp.broadinstitute.org/hapdip/GIAB/P.vcf.gz

## Benchmarking using hapdip (Li, 2015)

# get hapdip

https://github.com/lh3/hapdip

# evaluate

k8 hapdip.js distEval -d 10 -b N+P.auto.bed.gz P.vcf.gz variants.vcf.gz

## Run 16GT

# get 16GT (version 1.0)

https://github.com/aquaskyline/16GT

# build genome reference

https://github.com/aquaskyline/16GT/blob/master/README.md

# convert bam to snapshot (takes about 1.5 hours)

bam2snapshot -i human_g1k_v37_decoy.fasta.index -b NA12878.mem.bam -o output/prefix

# call variants (takes about 0.5 hour)

snapshotSnpcaller -i human_g1k_v37_decoy.fasta -o output/prefix

perl txt2vcf.pl output/prefix.txt NA12878 human_g1k_v37_decoy.fasta > variants.vcf

# filter variants

# get dbSNP138 for hs37d5

wget ftp://gsapubftp-anonymous@ftp.broadinstitute.org/bundle/b37/dbsnp_138.b37.vcf.gz

# filter

perl filterVCF.pl variants.vcf dbsnp_138.b37.vcf.gz > variants.filtered.vcf

# result

variants.filtered.vcf

## Run GATK UnifiedGenotyper

# get GATK (version 3.6-0-g89b7209)

https://software.broadinstitute.org/gatk/

# call variants

java -Xms1G -Xmx32G -jar GenomeAnalysisTK-3.6.jar -T UnifiedGenotyper -R human_g1k_v37_decoy.fasta -I NA12878.mem.bam -stand_emit_conf 10 -stand_call_conf 30 -nt 36 -o NA12878.vcf --genotype_likelihoods_model BOTH

# filter variants as suggested by (Li, 2015)

k8 hapdip.js deovlp NA12878.vcf | k8 hapdip.js upd1gt | k8 hapdip.js anno > NA12878.anno.vcf

k8 hapdip.js filter NA12878.anno.vcf > NA12878.filtered.vcf

# result

NA12878.filtered.vcf

## Run GATK HaplotypeCaller

# get GATK (version 3.6-0-g89b7209)

https://software.broadinstitute.org/gatk/

# call variants

java -Xms1G -Xmx32G -jar GenomeAnalysisTK-3.6.jar -T HaplotypeCaller -R human_g1k_v37_decoy.fasta -I NA12878.mem.bam -stand_emit_conf 10 -stand_call_conf 30 -nct 36 -o NA12878.vcf

# filter variants as suggested by (Li, 2015)

k8 hapdip.js deovlp NA12878.vcf | k8 hapdip.js upd1gt | k8 hapdip.js anno > NA12878.anno.vcf

k8 hapdip.js filter NA12878.anno.vcf > NA12878.filtered.vcf

# result

NA12878.filtered.vcf

## Run Freebayes

# get freebayes (v0.9.21-7-g7dd41db)

https://github.com/ekg/freebayes

# call variants

/work-zfs/mschatz1/rb/freebayes/scripts/fasta_generate_regions.py human_g1k_v37_decoy.fasta 1000000 > human_g1k_v37_decoy.region

freebayes/scripts/freebayes-parallel human_g1k_v37_decoy.region 36 -f human_g1k_v37_decoy.fasta NA12878.mem.bam > NA12878.vcf

# filter variants as suggested by (Li, 2015)

k8 hapdip.js deovlp NA12878.vcf | k8 hapdip.js upd1gt | k8 hapdip.js anno > NA12878.anno.vcf

k8 hapdip.js filter NA12878.anno.vcf > NA12878.filtered.vcf

# result

NA12878.filtered.vcf

## Run Fermikit

# get fermikit (version 0.13)

https://github.com/lh3/fermikit

# get fermi (version 1.1-r751-beta)

https://github.com/lh3/fermi.git

# extract reads from bam

samtools fastq -1 NA12878.1.fq -2 NA12878.1.fq NA12878.mem.ns.bam

gzip NA12878.1.fq

gzip NA12878.2.fq

# run fermi to assemble the reads

# notice: fermikit suggests using fermi2 for assembly. However, we failed to run this data on fermi2, it was taking too long (5 days) and generated no output at the final step called “simplify”. So in order to benchmark fermikit, we reverted to using fermi. As suggested by the author Heng Li, this will lead to worse performance.

fermi/run-fermi.pl -Pe fermi/fermi -t 36 -p NA12878.fermi1 NA12878.1.fq.gz NA12878.2.fq.gz > fermi1.mak

make -j 36 -f fermi1.mak

# run variant calling

fermi.kit/run-calling -t36 human_g1k_v37_decoy.fasta NA12878.fermi1.p1.mag.gz > call.sh

sh call.sh

# result

NA12878.fermi1.p1.flt.vcf.gz

## Run ISAAC

# get ISAAC (version 2.0.13)

https://github.com/Illumina/isaac2

# call variant

for i in {1..22} X Y; do isaac_variant_caller/bin/starling2 -bam-file NA12878.mem.bam --gvcf-file NA12878.$i.gvcf -bam-seq-name $i -samtools-reference human_g1k_v37_decoy.fasta -genome-size 3000000000 &\true; done

wait

# generate vcf from gvcf

# create header

grep '^#' NA12878.Y.gvcf >> NA12878.vcf

# concatenate results

for i in {1..22} X Y; do grep -v '^#' NA12878.$i.gvcf | awk '$5!="."' >> NA12878.vcf; done

# result

NA12878.vcf

## Run VarScan2

# get VarScan2 (version 2.4.3)

http://dkoboldt.github.io/varscan/

# call variant

for i in {1..22} X Y; samtools mpileup -r $i -f human_g1k_v37_decoy.fasta NA12878.mem.bam | java -jar VarScan.v2.4.3.jar mpileup2cns --output-vcf --variants 1>varscan_$i.vcf &\true; done

wait

# concatenate results

vcfcat varscan_{1..22}.vcf varscan_{X,Y}.vcf > NA12878.vcf

# result

NA12878.vcf

## Download Illumina Omni 2.5 and Evaluate SNPs (Genomes Project, et al., 2012)

# download

wget ftp://ftp.1000genomes.ebi.ac.uk/vol1/ftp/release/20130502/supporting/hd_genotype_chip/ALL.chip.omni_broad_sanger_combined.20140818.snps.genotypes.vcf.gz

# extract NA12878 (using vcftools (Danecek, et al., 2011) and bedtools (Quinlan and Hall, 2010))

vcf-subset –r –e –c NA12878 ALL.chip.omni_broad_sanger_combined.20140818.snps.genotypes.vcf.gz > NA12878.omni.vcf

# extract false positive SNPs

k8 hapdip.js distEval -d 10 -s distEval -b N+P.auto.bed.gz P.vcf.gz variant.vcf 1>fpfn

awk '$5=="FP"&&$4==0' fpfn > snpfp.bed

bedtools intersect -a variant.vcf -b snpfp.bed | awk '!/^#/&&(length($4)==1&&length($5)==1)' > snpfp.vcf

# show matches and mismatches

perl -e '%a=();open $b,"NA12878.omni.vcf" or die $!;while(<$b>){chomp;@f=split;$key="$f[0]-$f[1]";$value="$f[3]-$f[4]";$a{$key}="$value";};open $c,"snpfp.vcf" or die $!;while(<$c>){chomp;@f=split;$key="$f[0]-$f[1]";$value="$f[3]-$f[4]";if(defined $a{$key}){print "$key\t$value\t$a{$key}\n";};}'

# Supplemental References

Danecek, P.*, et al.* The variant call format and VCFtools. *Bioinformatics* 2011;27(15):2156-2158.

Genomes Project, C.*, et al.* An integrated map of genetic variation from 1,092 human genomes. *Nature* 2012;491(7422):56-65.

Li, H. FermiKit: assembly-based variant calling for Illumina resequencing data. *Bioinformatics* 2015:btv440.

Quinlan, A.R. and Hall, I.M. BEDTools: a flexible suite of utilities for comparing genomic features. *Bioinformatics* 2010;26(6):841-842.

Zook, J.M.*, et al.* Integrating human sequence data sets provides a resource of benchmark SNP and indel genotype calls. *Nat Biotechnol* 2014;32(3):246-251.
