# Supplementary material for: 16GT: a fast and sensitive variant caller using a 16-genotype probabilistic model
Source: Gigascience. 2017 Jun 15;6(7):1–4. doi: 10.1093/gigascience/gix045 (PMC5570013; doi:10.1093/gigascience/gix045)
Supplement: Response-to-Reviewer-Comments_Original-Submission.pdf [file gix045_Response-to-Reviewer-Comments_Original-Submission.pdf]

## Author's Response To Reviewer Comments

Please check submitted a response letter.

## View Letter

Date: 15 May 2017

To: "Ruibang Luo" aquaskyline@gmail.com,rluo5@jhu.edu

From: "GigaScience Editorial Office" editorial@gigasciencejournal.com

Subject: Your submission to GigaScience - GIGA-D-17-00091

GIGA-D-17-00091
16GT: a fast and sensitive variant caller using a 16-genotype probabilistic model
Ruibang Luo, Ph.D.; Michael C Schatz; Steven L Salzberg
GigaScience

Hi Ruibang,

Your manuscript "16GT: a fast and sensitive variant caller using a 16-genotype probabilistic model" (GIGA-D-17-00091) has been assessed by our reviewers. Based on these reports, and my own assessment as Editor, I am pleased to inform you that it is potentially acceptable for publication in GigaScience, once you have carried out some essential revisions suggested by our reviewers.

One referee flags the comparisons you make, so please make sure there is sufficient comparisons and citation of the state-of-the-art in this field (e.g. its been highlighted on the pre-print that Scalpel, VarScan2, VarDict, Mutect2 and Strelka have not been included as benchmarks).

Their reports, together with any other comments, are below. Please also take a moment to check our website at http://giga.edmgr.com/ for any additional comments that were saved as attachments.

We are also interested in using the code in this paper to test out CodeOcean (https://codeocean.com/) as we thought it would be a nice example to test the potential of this platform. If we have any questions or if you have any questions for us we'll be in touch.

Once you have made the necessary corrections, please submit a revised manuscript online at:

http://giga.edmgr.com/

If you have forgotten your username or password please use the "Send Login Details" link to get your login information. For security reasons, your password will be reset.

Please include a point-by-point within the 'Response to Reviewers' box in the submission system. Please ensure you describe additional experiments that were carried out and include a detailed rebuttal of any criticisms or requested revisions that you disagreed with. Please also ensure that your revised manuscript conforms to the journal style, which can be found in the Instructions for Authors on the journal homepage.

The due date for submitting the revised version of your article is 13 Aug 2017.

We look forward to receiving your revised manuscript soon.

Best wishes,

Scott Edmunds, Ph.D.
GigaScience
www.gigasciencejournal.com

Reviewer reports:
Reviewer #1: The authors present a new model that can call both SNPs and INDELs by expanding the number of possible allele states to 16. The paper is well written, the model is an interesting contribution, and the results are compelling. I would like to see a little more detail in a few sections of the paper.

The standard method for communicating the true positive / false negative trade off in variant calling is a ROC-style line plot. The shape of this curve can be insightful for readers who need place their experiments at different points along this plot depending on the particulars of their experiment. Since table 2 only reports a single point on that curve, the readers do not have this context. It is also not clear that these numbers represent comparable points along their curves.

I don't understand why the proportion of false positives in dbSNP v138 is interesting when calling against NA12878 and why having a higher proportion in dnSNP v183 is better. I recognize that these are polymorphic sites, but what about that property is relevant to this analysis?

The model has several "empirically defined" parameters. It would be nice to describe this analysis so that users could modify the parameters for their own experiments. For example, the model will need to be retuned for long reads.

16GT does not appear to support multi-sample calling. I think the model presented here is good, but

unless the software can handle many samples, or at least produce a GVCF, it may see little use.

- Ryan Layer, University of Utah

Reviewer #2: Luo, R. etc described a new 16GT variant caller optimized for Illunima sequencing data that uses a new 16-genotype probabilistic model to unify SNP and indel calling. They demonstrated the improved sensitivity for SNPs and comparable accuracy for indels comparing to GATK HaplotypeCaller, using genome of NA12878 in GIAB project. 16GT more comprehensively models 16 genotypes to unify SNP and indel calling in the same algorithm. 16GT appears to be a useful alternative tool for analyzing germline sequencing using Illumina platform.

A few comments:

1. Need to emphasize that at least at the moment, 16GT can only be applied to germline sequencing using Illumina sequencing platform, and not appropriate for cancer genome sequencing, especially clinical cancer samples, where tumor cellularity varies greatly and not fit those models.
2. Can authors comment on whether increased sensitivity of SNPs is due to incorporation of indels into the model, or are those additional SNPs called have indel as the 2nd allele?
3. Can authors discuss the limitations of 16GT? What's the indel size limit? Should sex chromosomes be treated differently if gender is known?
4. I'm not keen to highlight better indel performance over GATK's UnifiedGenotyper, as it's known to be not a good indel caller, and not widely used for indels nowadays.
5. Given the run time in Table 2, I'm not sure "16GT ran faster" should be in the abstract.

--

Please also take a moment to check our website at for any additional comments that were saved as attachments. Please note that as GigaScience has a policy of open peer review, you will be able to see the names of the reviewers.
