# Supplementary material for: 16GT: a fast and sensitive variant caller using a 16-genotype probabilistic model
Source: Gigascience. 2017 Jun 15;6(7):1–4. doi: 10.1093/gigascience/gix045 (PMC5570013; doi:10.1093/gigascience/gix045)
Supplement: Reviewer-1-Report-(Original-Submission).pdf [file gix045_Reviewer-1-Report-(Original-Submission).pdf]

Reviewer Report

Title: 16GT: a fast and sensitive variant caller using a 16-genotype probabilistic model

Version: Original Submission **Date:** 5/8/2017

Reviewer name: Ryan Layer

Reviewer Comments to Author:

The authors present a new model that can call both SNPs and INDELs by expanding the number of possible allele states to 16. The paper is well written, the model is an interesting contribution, and the results are compelling. I would like to see a little more detail in a few sections of the paper.The standard method for communicating the true positive / false negative trade off in variant calling is a ROC-style line plot. The shape of this curve can be insightful for readers who need place their experiments at different points along this plot depending on the particulars of their experiment. Since table 2 only reports a single point on that curve, the readers do not have this context. It is also not clear that these numbers represent comparable points along their curves.I don't understand why the proportion of false positives in dbSNP v138 is interesting when calling against NA12878 and why having a higher proportion in dnSNP v183 is better. I recognize that these are polymorphic sites, but what about that property is relevant to this analysis?The model has several "empirically defined" parameters. It would be nice to describe this analysis so that users could modify the parameters for their own experiments. For example, the model will need to be retuned for long reads.16GT does not appear to support multi-sample calling. I think the model presented here is good, but unless the software can handle many samples, or at least produce a GVCF, it may see little use.- Ryan Layer, University of Utah

Level of Interest

Please indicate how interesting you found the manuscript: An article whose findings are important to those with closely related research interests

Quality of Written English

Please indicate the quality of language in the manuscript: Acceptable

Declaration of Competing Interests

Please complete a declaration of competing interests, considering the following questions:

- Have you in the past five years received reimbursements, fees, funding, or salary from an organisation that may in any way gain or lose financially from the publication of this manuscript, either now or in the future?
- Do you hold any stocks or shares in an organisation that may in any way gain or lose financially from the publication of this manuscript, either now or in the future?

- Do you hold or are you currently applying for any patents relating to the content of the manuscript?
- Have you received reimbursements, fees, funding, or salary from an organization that holds or has applied for patents relating to the content of the manuscript?
- Do you have any other financial competing interests?
- Do you have any non-financial competing interests in relation to this paper?

If you can answer no to all of the above, write 'I declare that I have no competing interests' below. If your reply is yes to any, please give details below.

I declare that I have no competing interests

I agree to the open peer review policy of the journal. I understand that my name will be included on my report to the authors and, if the manuscript is accepted for publication, my named report including any attachments I upload will be posted on the website along with the authors' responses. I agree for my report to be made available under an Open Access Creative Commons CC-BY license (http://creativecommons.org/licenses/by/4.0/). I understand that any comments which I do not wish to be included in my named report can be included as confidential comments to the editors, which will not be published.

I agree to the open peer review policy of the journal
