# Supplementary material for: 16GT: a fast and sensitive variant caller using a 16-genotype probabilistic model
Source: Gigascience. 2017 Jun 15;6(7):1–4. doi: 10.1093/gigascience/gix045 (PMC5570013; doi:10.1093/gigascience/gix045)
Supplement: Reviewer-2-Report-(Original-Submission).pdf [file gix045_Reviewer-2-Report-(Original-Submission).pdf]

Reviewer Report

Title: 16GT: a fast and sensitive variant caller using a 16-genotype probabilistic model

Version: Original Submission **Date:** 5/15/2017

Reviewer name: Zhongwu Lai

Reviewer Comments to Author:

Luo, R. etc described a new 16GT variant caller optimized for Illunima sequencing data that uses a new 16-genotype probabilistic model to unify SNP and indel calling. They demonstrated the improved sensitivity for SNPs and comparable accuracy for indels comparing to GATK HaplotypeCaller, using genome of NA12878 in GIAB project. 16GT more comprehensively models 16 genotypes to unify SNP and indel calling in the same algorithm. 16GT appears to be a useful alternative tool for analyzing germline sequencing using Illumina platform.A few comments:1. Need to emphasize that at least at the moment, 16GT can only be applied to germline sequencing using Illumina sequencing platform, and not appropriate for cancer genome sequencing, especially clinical cancer samples, where tumor cellularity varies greatly and not fit those models.2. Can authors comment on whether increased sensitivity of SNPs is due to incorporation of indels into the model, or are those additional SNPs called have indel as the 2nd allele?3. Can authors discuss the limitations of 16GT? What's the indel size limit? Should sex chromosomes be treated differently if gender is known?4. I'm not keen to highlight better indel performance over GATK's UnifiedGenotyper, as it's known to be not a good indel caller, and not widely used for indels nowadays.5. Given the run time in Table 2, I'm not sure "16GT ran faster" should be in the abstract.

Level of Interest

Please indicate how interesting you found the manuscript: An article whose findings are important to those with closely related research interests

Quality of Written English

Please indicate the quality of language in the manuscript: Acceptable

Declaration of Competing Interests

Please complete a declaration of competing interests, considering the following questions:

- Have you in the past five years received reimbursements, fees, funding, or salary from an organisation that may in any way gain or lose financially from the publication of this manuscript, either now or in the future?
- Do you hold any stocks or shares in an organisation that may in any way gain or lose financially from the publication of this manuscript, either now or in the future?
- Do you hold or are you currently applying for any patents relating to the content of the manuscript?

- Have you received reimbursements, fees, funding, or salary from an organization that holds or has applied for patents relating to the content of the manuscript?
- Do you have any other financial competing interests?
- Do you have any non-financial competing interests in relation to this paper?

If you can answer no to all of the above, write 'I declare that I have no competing interests' below. If your reply is yes to any, please give details below.

I have developed an NGS variant caller, VarDict, for cancer research and was published in Nucleic Acids Research in 2016.

I agree to the open peer review policy of the journal. I understand that my name will be included on my report to the authors and, if the manuscript is accepted for publication, my named report including any attachments I upload will be posted on the website along with the authors' responses. I agree for my report to be made available under an Open Access Creative Commons CC-BY license (http://creativecommons.org/licenses/by/4.0/). I understand that any comments which I do not wish to be included in my named report can be included as confidential comments to the editors, which will not be published.

I agree to the open peer review policy of the journal
